# Towards Interoperability in Clinical Research - Enabling FHIR on the Open-Source Research Platform XNAT

**DOI:** 10.1007/s10916-020-01600-y

**Published:** 2020-07-09

**Authors:** Maryna Khvastova, Michael Witt, Andrea Essenwanger, Julian Sass, Sylvia Thun, Dagmar Krefting

**Affiliations:** 1 University of Applied Sciences Berlin, Center of Biomedical Image and Information Processing, Ostendstraße 25, 12459 Berlin, Germany; 2 Berlin Institute of Health (BIH), Anna-Louisa-Karsch-Str. 2, 10178 Berlin, Germany; 3 Department of Medical Informatics, University Medical Center Göttingen, Von-Siebold-Straße 3, 37075 Göttingen, Germany

**Keywords:** FHIR standards framework, Patient resource, Medical data management, XNAT, System interoperability, REST architecture style

## Abstract

This paper presents an approach to enable interoperability of the research data management system XNAT by the implementation of the HL7 standards framework Fast Healthcare Interoperability Resources (FHIR). The FHIR implementation is realized as an XNAT plugin (Source code: https://github.com/somnonetz/xnat-fhir-plugin), that allows easy adoption in arbitrary XNAT instances. The approach is demonstrated on patient data exchange between a FHIR reference implementation and XNAT.

## Introduction

Interoperability is one of the key issues in research, as defined by the FAIR guiding principles for scientific data management [8]. This capability implies, in particular, the employment of common standards for data and metadata, that holds also for the clinical domain [5].

Although the medical imaging standard DICOM^1^ is well established in the clinical domain both in healthcare and biomedical imaging research, there is no such de-facto standard for patient data and electronic case report forms. While in healthcare HL7^2^ is increasingly used for communication, EHR-based data sharing, analytics, machine learning and more,^3^ the definition of self-defined study-specific data models is still common in research. CDISC^4^ is used with some extend for medical research but has never reached the level of a de-facto standard for others than clinical trials for regulatory approval [6]. OpenEHR^5^ is an alternative approach, focused on a common description of electronic health records [7]. In particular in academic medical research, where study subjects have also a treatment relationship with the institution, the current interoperability issue between healthcare and research is error-prone and laborious, and is the main barrier for the employment of existing data resources for research. Therefore, a common communication standard is of high interest to enable seamless data sharing between different systems. With the support of the REST architecture in HL7-FHIR,^6^ interoperability at the communication level is now becoming easier to achieve, as REST APIS are used in many research platforms and modern data management systems (DMS) and are often extensible. One example is the Extensible Neuroimaging Archive Toolkit XNAT, an open-source imaging informatics platform developed by the Neuroinformatics Research Group at Washington University [4]. It is widely used in neuroimaging research and related domains, such as biosignal processing [1]. As the name indicates, it is specifically designed to be extended by arbitrary data models and formats. With the latest release (XNAT 1.7), both the REST-API and the plugin capabilities have been redesigned to allow easy integration of custom extensions of the REST-API. It is, therefore, a promising candidate for the integration of the FHIR-API into the platform. In this manuscript, a generic solution is proposed to integrate the HL7-FHIR standard into XNAT. The approach is demonstrated on the built-in data type for the study subject, as it is strongly interwoven into the XNAT system and the least flexible data type. The solution is validated by patient data exchange with a FHIR reference implementation server HAPI-FHIR.^7^

## Methods

In this section, a brief overview of the employed features and techniques of the XNAT platform and the HL7-FHIR standard is given.

### XNAT

XNAT has been designed for neuroimaging research and therefore follows the logics of clinical research studies. The core data types in XNAT are *projects*, *subjects* and *experiments*, typically in a hierarchical relationship. A wide variety of built-in data types are available, ranging from different imaging modalities to provenance and quality assurance support. The data types are described within the xdat and xnat XML schemas. For new data models, various abstract data types are available, that can be extended by the definition of a new data type derived from the built-in type in an additional XML schema. Such data types are implemented as XNAT plugins, that are automatically integrated when present at startup. To a limited extent, built-in data types can also be extended with new elements manually in the user interface. Another important feature is the comprehensive REST-API offered by XNAT. This can be used to communicate remotely with the instance, from another system or a custom user interface. The new REST API available in XNAT 1.7 employs the capabilities of the *Spring* web-framework. It allows for the definition of custom REST endpoints for reading, writing, updating and deleting data and metadata, again to be provided as a plugin. The Spring framework performs the mapping of URLs and HTTP-methods to Java methods at runtime. Submitted data in the JSON-format is automatically transformed into equivalent Java objects accessible during request handling. Result objects are also converted into JSON-formatted data. XNAT itself is implemented in Java and plugins are built with the *gradle* build system. A basic configuration like the plugin version, library dependencies and additional build steps that are required to create a plugin from Java source code are described in the build.gradle file. The build system ensures that all requirements are collected before the compilation process is started and the plugin jar file is built. If the plugin file is placed in the respective folder within the XNAT directory structure, it is automatically integrated into the instance on upstart.

### HL7-FHIR

Fast Healthcare Interoperability Resources (FHIR) is a standard framework created by HL7. FHIR defines a set of health data resources and a REST API on top for accessing them. The FHIR specification is openly available under the creative commons license. Its content is freely available to implementers, architects and clinicians. The main building blocks of FHIR are so-called *Resources*. Resources are modular and extensible discrete semantic units. These resources represent clinical concepts, such as Patient, Diagnosis or Medication. Resources are instantiated in XML or JSON. A resource instance is expected to have an identifying URL, metadata, a human-readable XHTML summary and the actual data that is captured. Each resource defines which specific data elements are part of its structure, their names, cardinalities and constraints. All resources should have a human-readable representation. Nonetheless, FHIR relies strongly on coded information. Therefore, FHIR supports the terminology binding of *code systems* and *value sets*. Code systems enable the use of external terminologies such as LOINC^8^ or SNOMED CT^9^. A value set is a group of coded values that may be used in a specific context. Additionally, resources can contain links to other resources, these are called *references*. Since clinical information systems have varying requirements, the FHIR specification leaves room for flexibility. That means resources can be adapted to more specific use cases. This can be implemented by setting constraints to the data elements and making use of FHIR’s *extension framework*. The action of constraining and/or extending these resources is called *profiling*. A definition of a resource or a profile on a resource is captured using a StructureDefinition. A StructureDefinition acts as a machine-readable blueprint and can be used for validation.

### Patient description in XNAT and FHIR

**Fig. 1:**
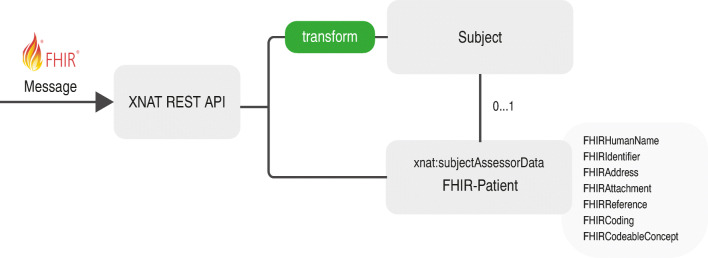
Architecture of the solution

Patient data has been chosen as the first data type to be enabled through a FHIR compatible interface in XNAT. These data is central in both healthcare and clinical research. However, due to the distinct domains, the focus is very different: in HL7, the core patient description is mainly administrative, encompassing contact details, spoken languages and a referring general practitioner. A corresponding profile defines 12 further data elements. In XNAT, the patient is considered a study subject, primarily described by its demographic data. It implies that XNAT subjects are often identified by a pseudonym and that they must always be part of a primary project, while FHIR has no mandatory data elements. Table 1 shows the core data elements of an HL7-FHIR patient and the corresponding XNAT subject elements. Further existing XNAT subject demographics elements, such as gestational_age, post_menstrual_age, birth_weight, are not shown; they would be modelled by other FHIR resources, such as *observations*. In the corresponding data elements, names and element types differ. In XNAT simple data types, e.g. string, float or date are used, while in FHIR there are many self-defined complex data types, such as HumanName with use, text, family, given, prefix, suffix and period (Fig. 1).

**Table 1:** Data elements describing a patient in XNAT and FHIR. Optional elements in the XNAT subject datatype are not shown

Description	XNAT	Type	FHIR	Type
Common elements				
Patient identifier	label	s	identifier	Identifier
Patient ID	subject_id	s	id	string
Patient’s name	name	s	name	HumanName
Date of birth	dob	d	birthDate	date
Gender	gender	s	gender	code
XNAT Mandatory				
Primary Project	project	p	–	–
FHIR only				
Patient record is in active use	–	–	active	boolean
Patient’s contact details	–	–	telecom	ContactPoint
Patient deceased or not	–	–	deceased	boolean
Patient’s address	–	–	address	Address
Patient’s marital status	–	–	maritalStatus	CodableConcept
Part of a multiple birth	–	–	multipleBirth	boolean or integer
Image of a patient	–	–	photo	Attachment
Contact party of the patient	–	–	contact	BackboneElement
Patient is animal	–	–	animal	BackboneElement
Patient’s languages	–	–	communication	BackboneElement
Patient’s care provider	–	–	generalPractitioner	Reference
Custodian organisation	–	–	managingOrganisation	Reference
Link to another patient	–	–	link	BackboneElement

Description: s: String data type, f: Float data type, d: Date, p: xnat:subjectData/project

### Validation

To validate the FHIR conformance, all public reference patient resources (n = 31) have been downloaded as JSON documents.^10^ They have been uploaded to both our XNAT instance as well as to a HAPI-FHIR server via curl, employing the respective REST-API endpoint Patient. After successful upload, the resources were downloaded through the REST-APIs and the resulting JSON documents were compared.

## Implementation

A dedicated plugin has been developed that provides the necessary data types and RESTful services.

### Data types

As both XNAT and HL7-FHIR rely on their respective names and data types, a mapping is required for the corresponding elements. Therefore the definition of a new REST endpoint is required, that translates these data elements. The new data elements can be directly implemented to be FHIR conformant. The main issue has been here the complex element types and the fact, that the subject data type can only be extended by string elements and data types derived from the subject are not properly integrated into the XNAT architecture. Therefore, a separate data type for the additional patient elements has been derived from the xnat:subjectAssessorData, an abstract data type for patient-related data. The FHIR-specific element types have been modelled as independent complex data types. All elements are implemented according to their cardinalities defined in FHIR specification. These data types were realized in an XSD-file. It contains the complex data types with their corresponding fields, relations and basic validation rules. The XNAT development libraries extend the gradle build process with custom hooks, that are used to generate Java classes for all data types described in the XSD file. This enables the developer to access items of the new data types through type-safe high-level Java classes. Persistence and retrieval of data items are managed through XNAT automatically.

### REST endpoints

Functions for each REST endpoint for reading, writing, updating and deleting patient information have been created and made available via code annotations. To build valid objects corresponding to the data types from the received JSON-data, several so-called *Services* have been implemented. Each data type has a corresponding service that handles data validation and object construction. The different services utilize each other because fields of certain FHIR data types reference other FHIR data types. Spring manages the presence of Service objects at all required places via dependency injection. All resources are assigned to a common project fhir, that was created in advance to fulfil the requirement of an existing primary project in XNAT.

## Results and discussion

All 31 patient resources could be successfully up- and downloaded in both the HAPI-FHIR server and the XNAT instance. The resulting JSON documents are identical except the assigned identifier. However, neither HAPI-FHIR nor our implementation currently processes and stores extensions. One exception occurred in the data schemes employed for the FHIR data types. The cardinality of relationships in the contact attribute of patient records could not be modelled as 0:n but only as 0:1. The plugin could be built, but XNAT could not load the plugin due to unknown restrictions in the XNAT framework. Again we assume a technical issue, as the problem only occurred in this specific data type. However, in none of the reference resources, more than one relationship was defined for a contact. So the issue might be of a minor practical impact.

It could be shown, that XNAT can be extended to handle FHIR messages and store the resource data correctly with the provided plugin framework. This constitutes a further step towards FAIR data management of the platform. The developed plugin can be directly used to allow patient data sharing with healthcare systems supporting HL7- FHIR, such as the HiGHmed platform. This open platform approach is part of the German Medical Informatics Initiative where nationwide data exchange is specified to be realized with FHIR; and where the adoption of terminologies such as SNOMED CT and LOINC is promoted, though rarely used in Germany at present [2]. The presented solution allows now for the seamless integration of medical imaging sources stored in XNAT into such an infrastructure by shared patient data. Prospectively, the XNAT plugin may be used as a template for the integration of further FHIR resources and would enable the integration of XNAT into arbitrary data-sharing frameworks using FHIR.

The process of creating the plugin took about six months, as all tools are open source, no further financial resources than staff costs were required. Mainly, knowledge of HL7-FHIR specialists and Java programmers were required. Although the XNAT data types and FHIR resources differ in their structure, it could be shown that mapping is feasible. The patient data case can be seen a particularly complex, as this data type is exceptionally deeply integrated into the XNAT data model, so it can be seen as a feasibility study for the full integration of FHIR into XNAT. The main difference in the information models are the explicit links in FHIR, that would be implicitly modelled in XNAT by inheritance of generic data types. Such hierarchies would be required to be modelled explicitly or mapped when translated to FHIR. But it constitutes no limitation of practical use.

We plan to extend the plugin to be able to process extensions and plan to redesign our existing data models for sleep research studies as FHIR resources.

The source code of this work is available at [3].

## Footnotes

1 https://www.dicomstandard.org

2 http://www.hl7.org/

3 http://www.hl7.org/implement/standards/product_brief.cfm?product_id=491

4 https://www.cdisc.org/

5 https://www.openehr.org/

6 https://www.hl7.org/fhir/

7 http://hapifhir.io/

8 http://loinc.org

9 http://snomed.info

10 http://hl7.org/fhir/2018Sep/downloads.html
